# Light Chain Restriction in Proximal Tubules—Implications for Light Chain Proximal Tubulopathy

**DOI:** 10.3389/fmed.2022.723758

**Published:** 2022-03-28

**Authors:** Maike Büttner-Herold, Nathalie Krieglstein, Teresa Chuva, Kaija Minuth, Frederick Pfister, Christoph Daniel, Monika Klewer, Anke Büttner, Fulvia Ferrazzi, Simone Bertz, Kerstin Amann

**Affiliations:** ^1^Department of Nephropathology, Institute of Pathology, Friedrich-Alexander-University Erlangen-Nuremberg, Erlangen, Germany; ^2^Department of Nephrology, Portuguese Institute of Oncology, Porto, Portugal; ^3^School of Psychology, College of Life and Environmental Sciences, University of Birmingham, Birmingham, United Kingdom; ^4^Institute of Pathology, Friedrich-Alexander-University Erlangen-Nuremberg, Erlangen, Germany

**Keywords:** renal tubule, light chain restriction, monoclonal gammopathies of renal significance, tubular crystals, cast nephropathy

## Abstract

Monoclonal gammopathy (MG) causes various nephropathies, which may suffice for cytoreductive therapy even in the absence of diagnostic criteria for multiple myeloma or B-cell non-Hodgkin lymphoma. The aim of this study was to better understand the significance of light chain (LC) restriction or crystals (LC-R/C) in proximal tubules in the spectrum of LC-induced nephropathies. A consecutive cohort of 320 renal specimens with a history of B-cell dyscrasia was characterized. Special attention was paid to immunohistochemical LC restriction in proximal tubules, tubular crystals or constipation, and ultrastructural findings. Complementary cell culture experiments were performed to assess the role of LC concentrations in generating LC restriction. Light chain restriction or crystals in proximal tubules was found in a quarter of analyzed cases (81/316) and was associated with another LC-induced disease in 70.4% (57/81), especially LC cast-nephropathy (cast-NP) and interstitial myeloma infiltration. LC restriction without significant signs of acute tubular injury was observed in 11.1% (9/81). LC-R/C was not associated with inferior renal function compared to the remainder of cases, when cases with accompanying cast-NP were excluded. Besides crystals, cloudy lysosomes were significantly associated with LC-R/C on an ultrastructural level. In summary, LC-R/C is frequent and strongly associated with cast-NP, possibly indicating that a high load of clonal LC is responsible for this phenomenon, supported by the observation that LC restriction can artificially be generated in cell culture. This and the lack of significant tubular injury in a subgroup imply that in part LC-R/C is a tubular trafficking phenomenon rather than an independent disease process.

## Introduction

In the past few years, “monoclonal gammopathy of renal significance (MGRS)” and especially its implications for prognosis and cytoreductive therapy have come into the focus of interest. In a large United States cohort, the prevalence of MG of undetermined significance (MGUS) was 4.2% with some renal diagnosis in 23% of cases (1). Renal disease in patients with MG or multiple myeloma (MM) is associated with an inferior prognosis (2), which can be improved by timely chemotherapeutic reduction of light chains (LCs) (3). Moreover, reduction of free LC in patients with MM is associated with better renal recovery, which associates with improved patient survival (4, 5).

Monoclonal gammopathy is associated with a variety of renal injuries involving the glomeruli, such as amyloidosis, monoclonal immunoglobulin deposition disease (MIDD), membranoproliferative glomerulonephritis (MPGN) or LC-induced C3 glomerulopathy, and also the tubular apparatus (6, 7), such as LC cast-nephropathy (cast-NP) (8). In the recent years, several studies focused on LC proximal tubulopathy (LCPT), which shows LC restriction, crystals, and/or signs of constipation in proximal tubular epithelial cells (9–12). Up-to-date, it is unclear whether LCPT is a clear-cut indication for cytoreductive therapy (7, 12) and whether LC restriction is a pathogenic event *per se*, indicating a genuine tubulopathy or whether it just indicates a trafficking/reabsorption mechanism (7, 13) due to high amounts of LC filtered in the glomerulus in MG. This causes insecurity in the daily routine of a nephropathologist, of whether to diagnose immunohistochemical findings associated with LCPT, namely, LC restriction or crystals (LC-R/C), as MGRS or to interpret it as an epiphenomenon of MG. Moreover, few systematic analyses on ultrastructural findings have been performed with regard to renal biopsies with LC restriction.

In this study, a consecutive cohort of patients with either a history of MG, MM, or other mature B-cell non-Hodgkin lymphoma (BNHL), or a diagnosis of LC-associated nephropathy in renal biopsy was evaluated to better understand the clinical and pathological context, in which LC restriction occurs. The aim of this study was to elucidate the significance of morphological findings typical of previously reported cases of LCPT, in particular LC restriction in proximal tubules in the setting of B-cell dyscrasia-induced nephropathies and to characterize associated ultrastructural findings.

## Materials and Methods

### Selection and Histological Assessment of the Cohort

Consecutive cases of a period of 23 months were retrieved from the files of the Department of Nephropathology, Friedrich–Alexander University Erlangen–Nüremberg, when fulfilling one or more of the inclusion criteria for B-cell dyscrasia (Supplementary Table 1): MG, MM, BNHL, and LC-associated nephropathy. In 35 patients, no clinical history of B-cell dyscrasia was reported, but biopsies were included, since a LC-associated nephropathy was diagnosed histologically. The retrospective analysis of archived renal biopsies was approved by the local ethics committee (reference number 4415).

Accordingly, 320 renal specimens (318 biopsies and 2 resection specimens) were selected from 315 patients and sections [stained with H&E and periodic acid–Schiff (PAS) reagent] were reevaluated by an experienced nephropathologist (MBH). Immunohistochemical stainings with antibodies specific for immunoglobulin A (IgA), immunoglobulin G (IgG), immunoglobulin M (IgM), C1q, C3 (all polyclonal, Dako, Glostrup, Denmark, Code No. IgA A0262, IgG A0423, IgM A0425, C1q A0136, and C3c A0062) were performed on formalin-fixed and paraffin-embedded (FFPE) material with current standard methods after digestion with protease from *Streptomyces griseus* (Sigma-Aldrich, Munich, Germany, Product No. P5147) on the Ventana Benchmark stainer (Basel, Switzerland). LC stainings were either performed with polyclonal rabbit antibodies supplied by Dako (Glostrup, Denmark, Code No. kappa A0192 and lambda A0194) or after 2013 antibody cocktails were used for detection of kappa (Dako, Glostrup, Denmark and Epitomics, Hannover, Germany, Cat. No. AC-0149) and lambda (Dako, Glostrup, Denmark, and Monosan, Am Uden, Netherlands, Cat. No. MONX10620) LC after antigen retrieval by heating with CC1 target retrieval buffer (Ventana, Basel, Switzerland, Cat. No. 950-124).

### Clinical Data

Clinical data was collected from files accompanying the renal biopsies. In two cases, MG was reported at a later time, both not showing signs of LC-R/C. Results of further hematologic workup after renal biopsy were not available. Categories of underlying disease (Supplementary Table 1) were established based on the diagnosis of mature BNHL (other than MM), MM, MG, or no established diagnosis (no information or mere suspicion of MG).

For proteinuria and hematuria, scoring systems were established (Supplementary Table 1) to achieve comparability between cases reported in different units. If glomerular filtration rate (GFR) was not reported, it was estimated using the Chronic Kidney Disease Epidemiology Collaboration (CKD-EPI) equation, whenever possible. Nephrotic syndrome was indicated as present, when reported or when the following criteria were met: serum albumin <30 mg/l, proteinuria >3.5 g/day (in 24-h urine or total protein-to-creatinine ratio on random urine specimen), and peripheral edema.

### Scoring of Histological Sections

Acute tubular injury (ATI) was scored semiquantitatively according to the percentage of cortical tubules with signs of ATI (0: no/minimal; 1: <25%; 2: 25 to <50%; 3: 50 to <75%; and 4: ≥75%). Intensity of LC staining in proximal tubular epithelial cells was scored semiquantitatively (0: negative; 1: mild; 2: moderate; and 3: strong reactivity). A significant predominance/restriction for a LC was postulated when ≥2 orders of intensity differed between the two LCs in the proximal tubular compartment; based on the observation that in unequivocal cases of LCPT with crystals or constipation, this difference was also repeatedly observed in the present cohort. Interstitial fibrosis/tubular atrophy (IF/TA) was rated in steps of 5%. Total numbers and numbers of globally sclerosed glomeruli were retrieved from the pathological reports.

### Evaluation of Semithin and Ultrathin Sections

In all the available cases, material processed for ultrathin sections (*n* = 275) was assessed by electron microscopy (EM) with particular attention to the presence of lobulated or angular lysosomes, lysosomes with mottled appearances, myelin bodies, cloudy lysosomes, lysosomes >2 μm in largest diameter, crystals, substructures in crystals, or fibrils in ultrathin sections (all as yes/no). Semithin section in cases with LC-R/C was evaluated for the presence of crystal or constipation (yes/no).

A total of 37 cases of native kidney biopsies without a known history of MG or other B-cell dyscrasia in the submitted files and without LC restriction in immunohistochemistry were selected as controls for EM findings.

### Cell Culture Experiments

Native human kappa (Biorad PHP280) and lambda (Biorad PHP281) LCs were labeled using CF598 and CF488 (Biotium 92,216 and 92,213) protein labeling kits according to manufacturer’s instructions. Efficiency of labeling was determined by measuring absorbance at 280, 488, and 590 nm and ranged between 0.7 and 0.9. Primary human proximal tubular epithelial cells (Cell Biologics, Chicago, United States) were plated on collagen-coated glass cover slides. On the next day, cells were stimulated with 5 or 50 μg/ml of κ LC-CF©598 and λ LC-CF©488 at ratios of 1:10, 10:10, or 10:1 in duplicates. After 24 h, cells were fixed, washed, and mounted in VECTASHIELD mount with 4’,6-diamidino-2-phenylindole (DAPI) (Vector Laboratories, Burlingame, United States). The experiment was repeated twice. Images were taken at 20X magnification [5 field of view (FOV) per slide] using a Zeiss LSM 710 confocal microscope and ZEN 2009 program (Carl Zeiss, Oberkochen, Germany). LC levels were determined as pixels above threshold relative to cell number (Fiji, U. S. National Institutes of Health, Bethesda, United States).

### Statistical Analysis

Statistical analyses were performed using Statistical Package for the Social Sciences (SPSS) for Windows (version 24 SPSS, IBM Incorporation, Munich, Germany). For the comparison of the five groups Kruskal–Wallis tests were used and *post hoc* testing with the Bonferroni correction. When two groups were compared, the Mann–Whitney *U* test was employed. For comparison of nominal parameters cross-tabulation, the chi-squared test was performed. If expected values in 2 × 2 cross-tabulation were <5, the Fisher’s exact test was chosen. *Post hoc* testing for cross-tabulation with more than 1 degree of freedom was performed, as published previously by Beasely et al. (14). Test results were considered as statistically significant, if *p*-values were <0.05.

## Results

### Patients’ Characteristics and Renal Diagnoses

Clinical characteristics, general histological findings, and diagnoses are shown in Table 1. A total of 320 tissue specimens of 315 patients were analyzed with an age range of 27.7–87.4 years (median: 69.2 years) and a gender distribution of 1:1.37 (female:male). Cast-NP was the most frequently diagnosed LC-associated nephropathy (*n* = 68), followed by AL amyloidosis (*n* = 58) and MIDD (*n* = 16). Interstitial myelomatous infiltration was found in seven renal specimens and infiltration by another mature BNHL was found in 12 renal specimens.

**TABLE 1:** Clinicopathological characteristics of the cohort.

Clinical/laboratory findings	
Sex (women/men)^a^	133/182
Age; median(min–max), n^a^	69.2 (27.7–87.4), 315
Diabetes mellitus (yes/no)^a^	70/196
Arterial hypertension (yes/no)^a^	170/86
Native/transplant specimens	308/12
GFR (ml/min); median(min–max), n	23.4 (1.2–119), 302
Serum creatinine (mg/dl); median(min–max), n	2.7 (0.5–18.3), 301
Proteinuria score; median(min–max), n	3 (0–4), 276
Hematuria score; median(min–max), n	1 (0–3), 236
Nephrotic syndrome (yes/no)	61/155
Reported disease; n: No/MG/MM/BNHL/MM and BNHL	35/169/75/39/2
Reported LC; n: Not reported/kappa/lambda/kappa and lambda/oligoclonal	150/91/72/6/1
**Histological findings**	
Number of glomeruli, median(min–max), n	14 (0–100), 320
Glomerulosclerosis%, median(min–max), n	14.3 (0–100), 313
IF/TA%, median(min–max), n	20 (0–100), 314
ATI Score, median(min–max), n	2 (0–4), 305
**Pathological diagnoses^b^**	
Cast-NP (n)	68+ suspicion of 1^c^
Amyloidosis (n)	
AL	58
AA	2
Unclear	6
MIDD (n)	16 + suspicion of 1^c^
Lymphoma infiltration (n)	
Myeloma	7
BNHL	12
Crystalglobulin NP (n)	1
Glomerunephritis (GN) (n)	
MPGN^d^	19
IgA-GN	16
GN with C3-deposition (infection-associated GN or C3-GP)	8 (5 C3-dominant)
Membranous GN	5
Pauci-immune crescentic GN	5
Immunotactoid GN	3
Fibrillary GN	1
Hypertensive and/or diabetic NP (n)	54
Podocytopathy^e^ (n)	14
FSGS of unclear origin (n)	3
TBMD (n)	3
ATI as main finding (n)	24
Thrombotic microangiopathy (n)	5
Interstitial nephritis (n)	9
Idiopathic nodular sclerosis	1
Insufficient material for diagnosis (n)	6

*n = number of analyzed cases.*

*^a^Exclusion of repeat biopsies.*

*^b^More than one diagnosis could be assigned per case.*

*^c^Suspicion of indicates that findings were insufficient for a definite diagnosis, e.g. single intratubular PAS-negative protein cylinders.*

*^d^immune-complex and cryoglobulinemic MPGN.*

*^e^Including minimal change glomerulopathy and primary FSGS.*

### Strong Association of LC-R/C With Interstitial Myelomatous Infiltration, Cast-NP, and a Clinical History of MM

Light chain restriction or crystals/constipation by light microscopy (LM) was found in 81 biopsies (78 with LC-R and 3 with crystals in EM, but no LC-R), further referred to as LC-R/C.

**FIGURE 1:**
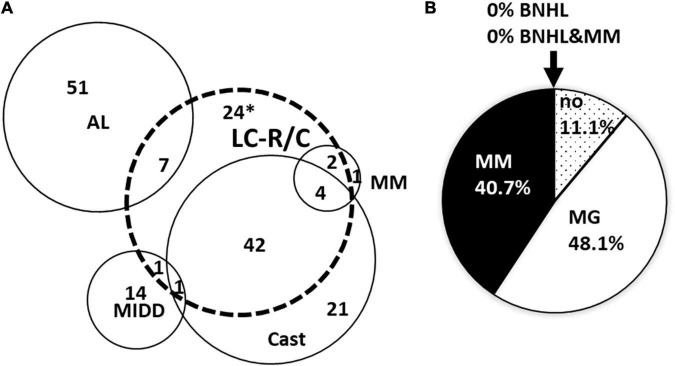
Overlap of light chain restriction or crystals (LC-R/C) with other LC-associated nephropathies and association with preexistent clinical diagnosis. **(A)** Strong overlap was observed between LC-R/C and cast-nephropathy (cast-NP) as well as interstitial myelomatous renal infiltration. Overlap with AL amyloidosis and monoclonal immunoglobulin deposition disease (MIDD) was much less conspicuous. *One case with suspicion of cast-NP is included in the 24 cases. **(B)** Association of the diagnosis of LC-R/C with clinical information on B-cell dyscrasia at the time of biopsy. In 48.1% of cases, MG was already known at the time of renal biopsy and in another 40.7% of cases, multiple myeloma was already diagnosed. No association with BNHL was observed. (no = MG not reported/only suspicion of MG; MG = monoclonal gammopathy, MM = multiple myeloma, BNHL = B-cell Non-Hodgkin lymphoma, MM&BNHL = multiple myeloma and B-cell Non-Hodgkin lymphoma).

Significant overlap existed of LC-R/C cases with other LC-related nephropathies (Figure 1A) with 70.4% (57/81) of LCPT cases being associated with another LC-associated nephropathy. Namely, 85.7% (6/7) of cases with interstitial myelomatous infiltration showed LC-R/C, 69.1% (47/68) of cases with cast-NP, and much smaller numbers of cases with AL amyloidosis (12.1%, 7/58) and MIDD (12.5%, 2/16). One case was associated with C3-dominant glomerulonephritis (GN), in which it was not to decide whether a LC-associated C3 glomerulopathy or infection-related GN was causative. No association of LC-R/C with any other GN or thrombotic microangiopathy, which has been described in the context of MG, was observed. No case of LC-R/C was accompanied by renal infiltration of a BNHL other than myeloma. The vast majority of LC-R/C cases was associated with a clinical history of MG (48.1%, 39/81) or MM (40.7%, 33/81), whereas no previous diagnosis of B-cell dyscrasia was reported in 11.1% (9/81). No association of LC-R/C with a history of BNHL or MM and BNHL was documented (Figure 1B).

The association of LC-R/C with renal function, pathology, and clinical findings depending on the presence or absence of cast-NP is given in Table 2. The association of LC-R/C with cast-NP was significant (*p* < 0.001) as was the association with MM infiltration in the kidney compared to cases with no lymphomatous or myelomatous infiltration (*p* = 0.002). Comparing all the cases with and without LC-R/C, cases with LC-R/C were associated with a history of MM compared to a history of MG (*p* = 0.001), inferior renal function (lower GFR and higher creatinine), and higher ATI scores (all *p* < 0.001). Except for the association with a history of MM compared to a history of MG (*p* < 0.001), this effect, however, completely vanished when excluding all the cases with cast-NP (all *p* > 0.05). Moreover, presence or absence of LC-R/C within the group of cast-NP had no influence on renal function (i.e., GFR, creatinine) or ATI (all *p* > 0.05) and was only significantly associated with a history of MM (*p* = 0.02), too.

**TABLE 2:** Association of cast-NP with light chain restriction or crystals (LC-R/C) and renal function.

	All cases^a^		*p*-Value	Cast-NP excluded^a^		*p*-Value	Cast-NP only^a^		*p*-Value
	No LC-R/C	LC-R/C		No LC-R/C	LC-R/C		No LC-R/C	LC-R/C	
Cast-NP yes/no	21/214	47/33	< 0.001	–	–	−	–	–	
InfiltrationMM/none	1/224	6/74	0.002	1/204	2/31	0.051	0/20	4/43	0.309
History of MG/MM	130/42	39/32	0.001	125/28	15/15	< 0.001	5/14	24/17	0.02
GFR (ml/min)^b^	26.4 (1.2;119) 226	13 (3;109)	< 0.001	28.3 (1.2;119) 206	23.6 (4.1;109)	0.367	10 (4.7;55)	10.1 (3; 70)	0.877
		73			30		20	43	
Creatinine (mg/dl)^b^	2.42 (0.6;11.8) 225	4.3 (0.5;18.3) 73	< 0.001	2.3 (0.6;10.7) 205	2.35 (0.5;12.05)	0.549	5.56 (1.6;11.8) 20	4.7 (1.04;18.3)	0.6
					30			43	
ATI score^b^	2 (0;4)	3 (0;4)	< 0.001	2 (0;4)	2 (0;4)	0.337	4 (2;4)	4 (2;4)	0.934
	224	79		203	32		21	47	

*^a^One cases with suspicion of Cast-NP was excluded from all analyses.*

*^b^Data indicated as median (range), n = number of cases analyzed.*

### Light Microscopy and Immunohistochemical Findings in Cases With LC-R/C

**FIGURE 2:**
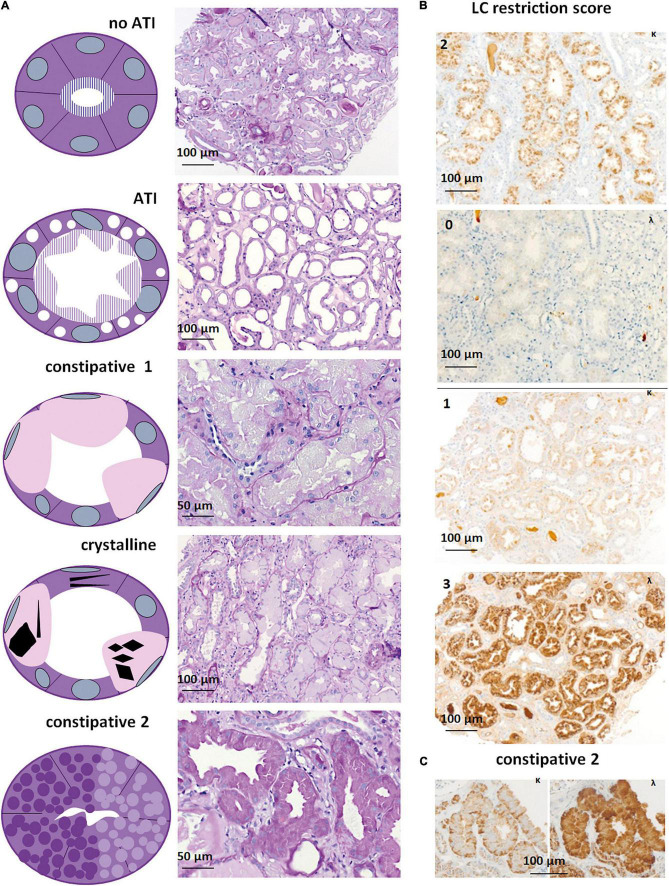
Light microscopy and immunohistochemistry of cases with LC-R/C. **(A)** Five patterns of LC-R/C were defined on morphological grounds, including the no acute tubular injury (ATI) group with LC restriction and no more than mild signs of ATI, the ATI group showing more than mild signs of ATI, the constipative type 1 (constipative1) group showing pale and distended epithelia, a group with crystalline inclusions, which in some cases was associated with constipation and a second constipative type 2 (constipative2) group, characterized by distended epithelia with prominent granulation [all the sections stained by periodic acid–Schiff (PAS) reaction]. **(B)** Examples of LC restriction in kappa (κ) and lambda (λ) immunohistochemistry. LC restriction was diagnosed when ≥2 orders of intensity difference were observed between the two light chains, i.e., score 0 and 2 (top) or 3 or score 1 and 3 (bottom). **(C)** LC restriction in a case of constipative type 2 LC-R/C showing a “flower”-like accentuation of the staining at the basolateral aspect of the involved epithelia. Light microscopic pictures were taken with an AxioCam MRc and an Axio Imager A1 microscope (Zeiss, Germany). Scale bars as indicated. κ = kappa-light chain immunohistochemistry, λ = lambda-light chain immunohistochemistry.

**FIGURE 3:**
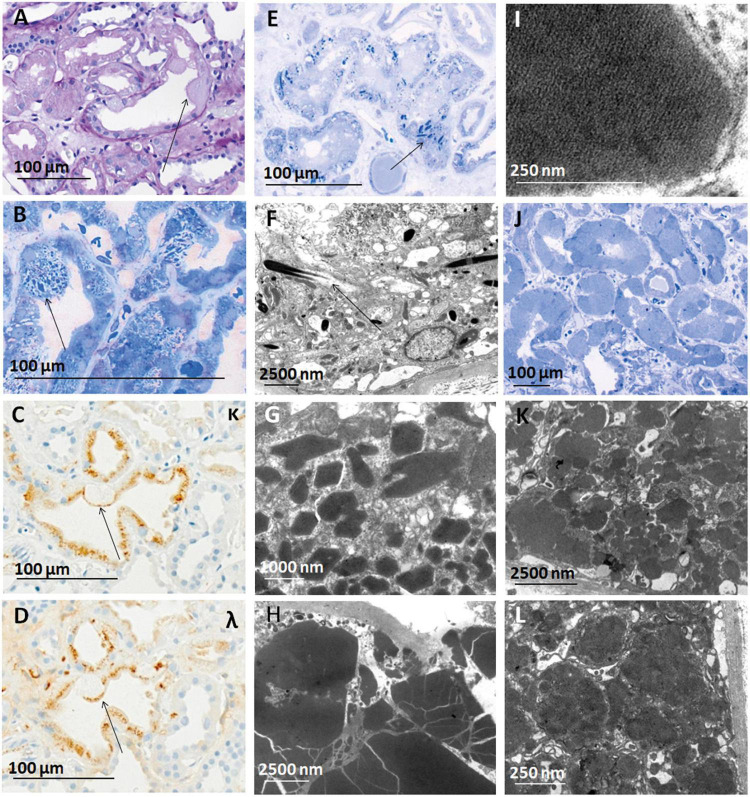
Constipation and crystals observed in cases of LC-R/C. **(A–D)** Crystalline LC-R/C characterized by a subgroup of proximal tubular epithelial cells constipated with PAS-negative material [**(A)**, arrow], which in toluidine blue-stained semithin sections shows intracytoplasmic crystals **(B)** and a negativity for both the light chain stainings in the constipated area of the epithelial cell [**(C,D)**, arrows], whereas apical protein resorption vacuoles show no LC restriction. **(E)** Toluidine blue-stained semithin section of a case with needle-shaped intraepithelial crystals and **(F)** needle-shaped crystals in electron microscopy. **(G)** Crystals of rhomboid and **(H)** polyhedric shapes, with the latter showing sharp edges and cracks in electron microscopy. **(I)** Lattice-like substructure in a cytoplasmic inclusion, which was also interpreted as a characteristic of the crystalline LC-R/C group. **(J)** Toluidine blue-stained semithin section of a case with tubular constipation showing ballooned epithelia filled with homogenous, pale intracytoplasmic material and nuclei dislocated to the cell borders **(K,L)**. In some cases, the constipated tubular epithelial cells were found to contain “cloudy,” amorphous material in their cytoplasm. Light microscopic pictures were taken with an AxioCam MRc and an Axio Imager A1 microscope (Zeiss, Germany). Electron microscopic pictures were taken with a Leo912 electron microscope (Zeiss, Germany). Scale bars as indicated. PAS, periodic acid-Schiff; EM, electron microscopy; κ, kappa-light chain immunohistochemistry; λ, lambda-light chain immunohistochemistry.

Out of 316 kidney specimens evaluated by LM (Figure 2A), LC immunohistochemistry (313, Figures 2B,C), and EM (275, Figure 3), 81 kidney specimens (25.6%) showed LC-R (Figures 2B,C), negativity for both the LCs in constipated epithelia (*n* = 1, Figures 3A–D) and/or cytoplasmic crystals by LM or EM (Figure 3), referred to as LC-R/C.

In our cohort, five different morphologic patterns of LC-R/C were identified by LM when taking into account morphological observations and previously published patterns of injury (9) (Figure 2A): (i) no ATI: ATI scores 0–1 (9/81, 11.1%); (ii) ATI: with signs of ATI (score ≥ 2) only (55/81, 67.9%); (iii) constipative type 1 (constipative1): ballooned tubular epithelia filled with pale amorphic material or PAS-negative or mildly positive material (3/81, 3.7%); (iv) crystalline: with intraepithelial crystals accompanied by abovementioned signs of constipation in 4 of 8 cases (8/81, 9.9%); and (v) constipative type 2 (constipative2): showing few or single tubules with prominent granulation (PAS variably positive) distending the epithelia with a “flower”-like accentuation at the basal aspect of the epithelia in LC immunohistochemistry (Figure 2C, 6/81, 7.4%). In the latter, in 1 of 6 cases, LC predominance was only restricted to the constipated tubules.

Looking at all the LC-R/C cases, LC restriction was lambda in 59.3% (48/81) and kappa in 37% (30/81) (Table 3). In three cases with cytoplasmic crystals (3.7%, 3/81), LC restriction was missing. In one of those cases, an atypical staining pattern was present, in the sense that both the kappa- and lambda-LC immunohistochemistry were negative in the constipated tubular epithelial cells filled with crystals (Figures 3A–D). In the other two cases, LM and LC immunohistochemistry were both unremarkable, with either no or only mild signs of ATI. However, in EM, single intraepithelial crystals were found in tubular epithelial cells, so that both the cases were included in the group of crystalline LC-R/C.

**TABLE 3:** Comparison of subdivided LC-R/C cases.

	All LC-R/C	noATI	ATI	Constipative1	Crystalline	Constipative2	*p*-value*	*p*-value, *post hoc*
Total number of biopsies (%)	81	9 (11.1%)	55 (67.9%)	3 (3.7%)	8 (9.9%)	6 (7.4%)		
**Clinical parameters:**								
Age at biopsy (years), mean (range)	69.3 (35.4–87.4)	61.9 (48.8–77.7)	69.1 (35.4–87.4)	76 (68.9–80.5)	71.4 (47.1–85)	78.3 (60.7–84.3)	0.156	
Sex (f/m)	36/45	3/6	25/30	0/3	4/4	4/2	0.383	
Diabetes mellitus (yes/no)	15/47	1/7	10/31	1/1	1/5	2/3	0.701	
Hypertension (yes/no)	38/23	3/4	25/16	2/0	4/2	4/1	0.544	
GFR ml/min	13.1 (3–109), 74	69.1 (29.9–109), 8	10.2 (3–70), 49	22.1 (10.1–24), 3	30 (3.8–85.2), 8	18 (9.6–38.3), 6	<0.001	noATI-ATI < 0.001
Serum creatinine mg/dl	4.3 (0.5–18.3), 74	1.14 (0.5–2.1), 8	4.7 (1–18.3), 49	2.7 (2.6–5.5), 3	2.14 (0.8–10.4), 8	3.8 (1.3–4.4), 6	<0.001	noATI–ATI < 0.001
Proteinuria score	3 (0–4), 68	3 (2–4), 8	3 (0–4), 44	3.5 (3–4), 2	2.5 (0–4), 8	4 (4–4), 6	0.064	
Nephrotic syndrome (yes/no)	6/39	1/5	1/28	0/1	0/5	4/0	<0.001	constipative 2 < 0.001
Hematuria score	1 (0-3), 53	1 (0-1), 7	1 (0-3), 35	1 (1-1), 2	1 (0-3), 6	1 (0–2), 3	0.630	
History of No/MG/MM/BNHL	9/39/33/0	0/4/5/0	6/27/22/0	0/1/2/0	0/5/3/0	3/2/1/0	0.110	
Unknown/κ/λ/κ &λ	36/18/26/1	1/1/7/0	27/11/17/0	1/2/0/0	2/4/1/1	5/0/1/0	κ vs λ:	
							0.51	
**Biopsy findings:**								
Glomerulosclerosis%	8.7 (0–83.9), 79	13.8 (0–41.8), 9	8.5 (0–83.9), 54	6.3 (0–56), 3	6.1 (0–38.1), 8	33.3 (0–50), 5	0.430	
IF/TA%	20 (0–80), 80	5 (0–30), 9	20 (0–80), 55	15 (15–30), 3	20 (0–80), 8	25 (15–60), 5	0.092	
ATI score	3 (0–4), 80	1 (0–1), 9	4 (2–4), 55	4 (2–4), 3	3 (0–4), 8	3 (1–4), 5	<0.001	noATI-ATI < 0.001
								noATI-constipative 1 0.042
LC restriction κ/λ (IHC)	30/48	1/8	21/34	3/0	4/1	1/5	0.014	n.s.
Infiltration: no/MM/BNHL	75/6/0	9/0/0	49/6/0	3/0/0	8/0/0	6/0/0	0.547	
Coexisting LC associated renal diagnoses	42 Cast	2 AL amyloidosis	35 Cast	2 Cast	5 Cast	4 AL amyloidosis	AL <0.001	Cast-NP:
	4 Cast and MM infiltrate		4 Cast and MM infiltrate	1 AL amyloidosis			Cast <0.001	ATI <0.001
	1 Cast and MIDD		1 Cast and MIDD				MIDD	noATI 0.001
	1 susp. of Cast		1 susp. of Cast				0.914	Constipative 2 0.03
	1 MIDD		1 MIDD					AL:
	2 MM infiltrate		2 MM infiltrate					ATI <0.001
	7 AL amyloidosis							Constipative2 <0.001

### Comparison of LC-R/C Subtypes With Regard to Clinical and Pathological Findings

Acute tubular injury type was by far the most prevalent subtype (55/81, 67.9%, Table 3). No significant differences were found between the five subgroups of LC-R/C with regard to age, gender distribution, and the occurrence of diabetes or arterial hypertension. Comparing no ATI, which was defined by the lack of significant ATI (ATI score 0 or 1), with the other groups, no ATI was associated with better renal function with regard to GFR and serum creatinine. This observation reached statistical significance when compared to ATI-type LC-R/C (both *p* < 0.001, Table 3). Constipative2 LC-R/C was significantly associated with the presence of nephrotic syndrome (*p* < 0.001). No significant differences between the groups were observed with regard to proteinuria and hematuria. The vast majority of LC-R/C cases were associated with a history of MG (39/81, 48.1%) or MM (33/81, 40.7%) at the time of biopsy (Table 3). In the constipative2 group only, 1/6 cases were associated with a history of MM, whereas in 50% (3/6), no diagnosis of B-cell dyscrasia was reported. This observation, however, was not significantly different from the other groups. In all the 81 LCPT, lambda LC by immunohistochemistry was more prevalent than kappa LC (kappa:lambda = 1:1.6); in the constipative1 (3:0) and crystalline group (4:1), kappa LC was predominant. In constipative2, the majority of cases were positive for lambda-LC (1:5). These associations did not reach statistical significance, presumably due to the relatively small numbers of cases (Table 3). According to the definition of the no ATI subgroup, ATI scores were lower in this group than in the other groups reaching significance for the comparison to ATI (*p* < 0.001) and constipative1 (*p* = 0.042, Table 3). The ATI group was significantly associated with the presence of cast-NP (*p* < 0.001) and no ATI and constipative2 with the absence of cast-NP (*p* = 0.001 and 0.03, respectively). Constipative2 was significantly related to the presence of amyloidosis (*p* < 0.001) and ATI to the absence of amyloidosis (*p* < 0.001, Table 3). ATI was the only group in which myelomatous infiltration in the kidney was observed, which was statistically not significant (Table 3). No significant differences were observed between the groups when considering renal scarring (glomerulosclerosis and IF/TA).

Two cases were included in the crystalline subgroup for the presence of only very few intracytoplasmic crystals in EM, but did not show LC restriction by immunohistochemistry. Both the cases had no significant signs of ATI (scores 0 and 1). One was associated with a better renal function than the other cases with crystalline LC-R/C with a GFR of 85.2 ml/min/1.73 m^2^ and a serum creatinine of 0.8 mg/dl.

### Ultrastructural Characteristics of Renal Specimens With LC-R/C

**FIGURE 4:**
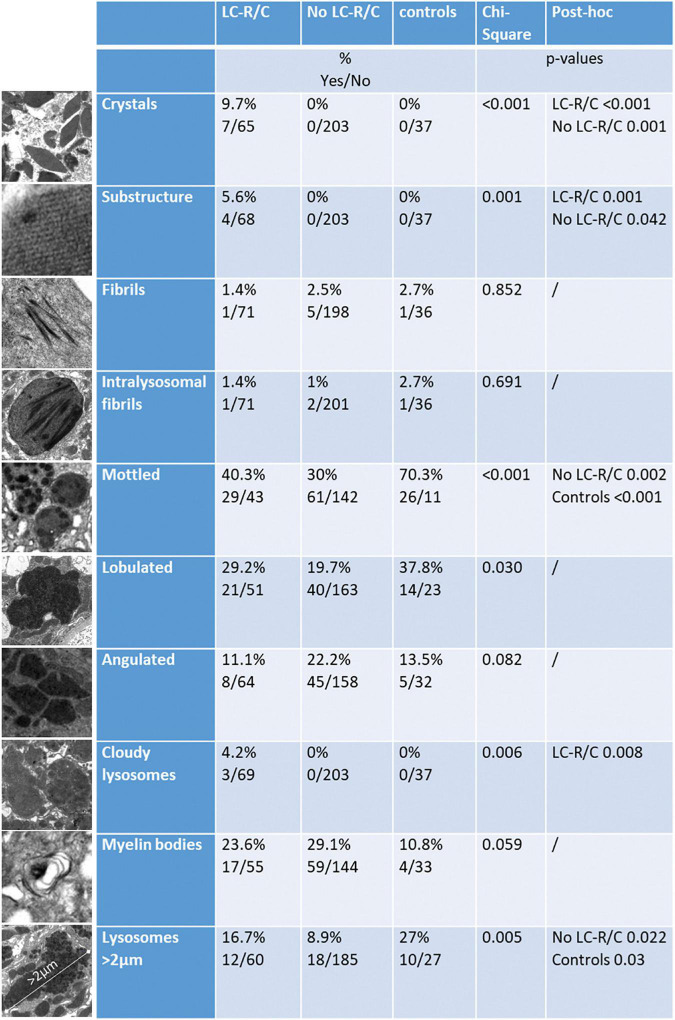
Comparison of cases with LC-R/C, without LC-R/C (no LC-R/C), and of controls unrelated to the cohort (controls) with regard to ultrastructural findings. At the left, examples of the analyzed ultrastructural finding are depicted. From top to bottom: crystalline inclusions; lattice-like substructure in an crystalline inclusion; fibrils (intracytoplasmic or intralysosomal); intralysosomal fibrils only, lysosomes with mottled appearance, lysosomes with lobulated or angulated contours, cloudy lysosomes, myelin bodies, and largest lysosome with a maximum diameter >2 μm. In the middle, the percentages of cases with the respective finding in the groups are indicated and below numbers of positive and negative cases (yes/no). At the right, results of cross-tabulation are indicated, showing significant differences.

In order to assess the ultrastructural findings associated with LC-R/C, ultrathin sections of all the cases of the cohort with available material were evaluated and findings were compared with those in the control cohort of 37 patients without reported history of B-cell dyscrasia and without LC restriction as assessed by immunohistochemistry. The study cohort was divided according to the presence or absence of signs of LC-R/C (Figure 4). The presence of intraepithelial fibrils, in general or exclusively intralysosomal, of lysosomes with mottled appearance or angulated or lobulated contours or of myelin bodies were not significantly associated with the presence of LC-R/C (Figure 4). In fact, lysosomes with mottled appearance were even more common in the control cases (*p* < 0.001) and least common in cases with no LC-R/C, but a history of B-cell dyscrasia (*p* = 0.002). As defined, crystals and substructures were only present in the LC-R/C group and in statistical analysis, they were significantly associated with LC-R/C (*p* < 0.001 and *p* = 0.001, Figures 3E–I, 4). Another feature significantly associated with LC-R/C was the presence of lysosomes filled with a cloudy, amorphous material (*p* = 0.008, Figures 3J–L, 4), a finding not observed in the no LC-R/C group or controls. Particularly, large lysosomes with a maximum diameter >2 μm were not a characteristic of LC-R/C, but were most frequent in the control cases (*p* = 0.03) and least common in the no LC-R/C cases (*p* = 0.022, Figure 4). When comparing the 37 controls with cases with LC-R/C, the presence of crystals and substructures (as defined) or cloudy lysosomes were only found in cases with LC-R/C. These differences did, however, not reach statistical significance (all *p* > 0.05). Differences were significant when comparing the LC-R/C cases with the no LC-R/C cases (crystals *p* < 0.001, substructures *p* = 0.004, and cloudy lysosomes *p* = 0.017).

### Induction of LC Predominance in Human Tubular Epithelial Cells by Variation of LC Ratios

**FIGURE 5:**
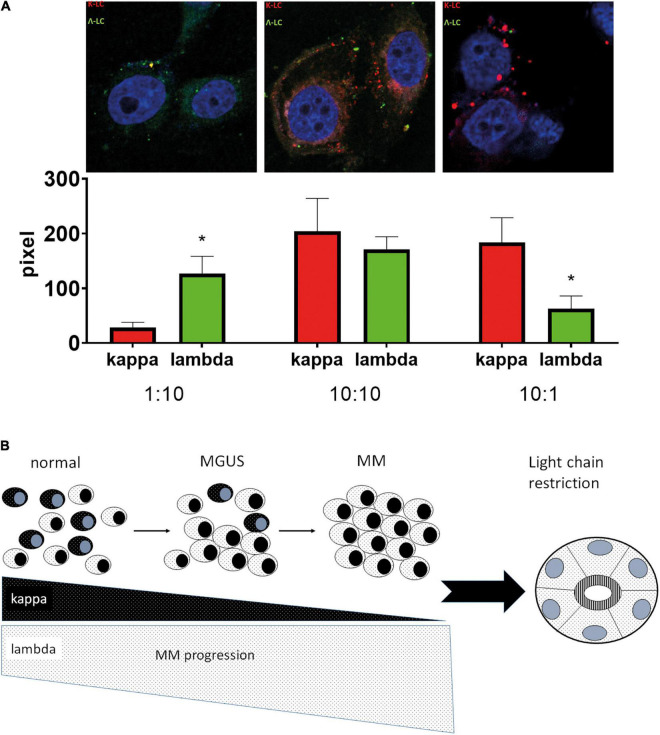
Hypothetical model of the development of LC restriction in the context of high monoclonal light chains. **(A)** After incubation of human proximal tubular epithelial cells with different ratios of labeled kappa- and lambda-LC, a predominance for the LC added in the higher concentration was observed in confocal microscopy, with significant differences at the ratio of kappa:lambda 1:10 (*p* = 0.049) and 10:1 (*p* = 0.001). Two independent experiments in duplicates were performed. Representative pictures were taken at 630X using a Zeiss710 confocal microscope. **(B)** Under physiological circumstances (normal), a mixture of kappa and lambda LC-positive plasma cells represents the pool of reactive plasma cells. During the course of the development of multiple myeloma (MM), a phase of MG of undetermined significance (MGUS) is passed, in which outgrowth of a plasma cell clone starts (here exemplified by lambda light chains). In the phase of MGUS, polyclonal plasma cells are still present and the plasma cell clone represents only a subgroup of the complete plasma cell pool, making a predominance for lambda LC in the serum, but no full-blown LC restriction. When the state of MM is reached, there is a strong expansion of the atypical plasma cell clone at the expense of normal plasma cells, which is accompanied by an increase of one LC and nearly complete loss of the other in the blood serum. This developmental process in myeloma genesis might explain the presence of LC restriction in cases with MM and high tumor load. *p < 0.05.

The significant association of LC-R/C with myeloma history and infiltration as well as cast-NP might indicate that high loads of one as compared to the other LC are relevant for the phenomenon of LC predominance/restriction in tubular epithelial cells. To test this hypothesis, we incubated primary human proximal tubular epithelial cells with fluorescence-labeled kappa- and lambda-LC at different ratios, to test whether a quantitative change in LC would be reflected in the intratubular storage of LC. Accordingly, the titration of kappa:lambda LC at ratios of 10:1; 10:10, and 1:10 leads to a predominance of the respective LC added at the higher concentration in the tubular epithelial cells after an incubation period of 24 h (Figure 5A), supporting the hypothesis that LC restriction can be generated by merely changing the relative quantity of LC.

## Discussion

In this study, renal specimens were analyzed with special emphasis on LC-R/C in proximal tubules, morphological and immunohistochemical parameters that have been described for the diagnosis of LCPT (9, 12). The presence of LC-R/C as a histological finding was set in the context of clinical and histological parameters and discussed in relation to previous reports of LCPT. The physiological fate of free LC in the kidney includes free filtration in the glomerulus and receptor-mediated reabsorption by proximal tubules (15), a very efficient but saturable process (15). LC are then degraded within lysosomes (16).

A quarter of the analyzed kidneys showed LC-R/C, making it a very frequent finding, as reported earlier for LCPT (9). In 70.4%, LC-R/C was associated with another LC-associated nephropathy, cast-NP in particular. Accordingly, LC in some cast-NP shows properties similar to cases with Fanconi syndrome (17) and proximal tubular changes in cast-NP resemble those in LC damage without cast-NP (18). An association of LCPT or tubular Fanconi syndrome with cast-NP and to a lesser extent AL amyloidosis has been reported earlier (17, 19–21), with one case preceding amyloidosis (22). 88.8% of cases with LC-R/C at biopsy had a history of MM (40.7%) or MG (48.1%) rather similar to another study, in which B-cell dyscrasia was diagnosed in 82.6% (12). Here, information on full hematological workup postbiopsy was not available. In a recent review (23), frequencies of 12–33% for associated MM and 61–80% for MGRS in LCPT were quoted and only very few other associated hematological diseases (1–2%) going in line with the lack of an association of LC-R/C with BNHL in our cohort.

Light chain restriction or crystals was associated with inferior renal function and higher ATI, which vanished after exclusion of cast-NP or looking at cast-NP alone. Cast-NP is known to be associated with high urinary excretion of pathologic LC and MM (24–27). In the course of progression of MG to MM, the percentage of clonal compared to normal plasma cells increases steadily (28), so that, hypothetically, the lack of one LC in proximal tubules might reflect the loss of the normal plasma cell pool (Figure 5B). This notion goes in line with our observation that LC predominance could be generated by changing the relative LC concentrations in cell culture. These findings imply that, at least in a subgroup, LC-R/C might not represent a genuine “tubulopathy,” but rather a sign of “physiological trafficking,” as suggested earlier (7, 11–13) in the context of high clonal LC loads. Another observation that could argue that LC-R is not always a disease *per se* is that in rat tubules only some human LC elicit tubulopathic effects (29). Accordingly, a subgroup of LC-R/C showed no or only mild signs of ATI as described earlier (11, 12) and was associated with better renal function, suggesting that at least in a subgroup LC are not tubulotoxic.

Some evidence, however, supports the notion of an independent disease process, including the frequent association of especially crystalline LCPT with tubular Fanconi syndrome (12, 19, 22) and, thus, a typical clinical picture. Furthermore, tubulotoxic effects of LC were shown in rats and cultured proximal tubular epithelial cells (15, 30–32). Moreover, patients with urinary high LC compared to non-specific proteinuria showed stronger proximal tubular damage (18).

Therefore, it is difficult to decide whether and when LC-R/C is an epiphenomenon or a genuine disease process and, thereby, an indication for cytoreductive therapy. In the vast majority of our cohort, an accompanying LC-associated nephropathy required therapy at any rate. In the remaining cases, LC-R/C may sometimes not suffice to justify a toxic therapy, so decision upon initiation of treatment probably has to be made on a case-by-case basis, as suggested earlier (7). Chemotherapy or stem cell transplantation for LCPT with Fanconi syndrome has been recommended (33) and can result in at least stable renal function (12). However, a proportion of untreated cases with crystalline LCPT also showed stable renal function (12). The frequent association of LC-R/C with LC nephropathies and MM, however, should warrant hematologic workup and close follow-up, especially as LCPT can precede another LC-induced nephropathy (22). Moreover, a thorough exclusion of another, not LC-associated nephropathy explaining the clinical findings must be performed.

In 2014, Herrera suggested different patterns of LCPT that overlap with our cohort (9). LCPT “without cytoplasmic inclusions” corresponds to our no ATI- and ATI-LC-R/C, although we and others (11) found a predominance of lambda-LC in contrast to the earlier report (9). The phenotypes “with cytoplasmic inclusions” and “with lysosomal ingestion/constipation” overlap with our crystalline and constipative1 subtypes and were also (though not significantly in our cohort) associated with kappa-LC and far less common than no ATI-/ATI-LC-R/C (9). In some earlier studies, crystalline LCPT was exclusively associated with kappa-LC (9, 12, 20). We found one case with single crystals associated with lambda-LC, which is also very rarely reported in literature (11). None of the cases included in our cohort, after thorough search, met the criteria of the acute tubulointerstitial nephritis variant of LCPT, as defined previously (9, 34). This goes in line with a cohort of LCPT published by Stokes et al. (12). In our cohort, only about 10% of cases showed LC reactivity at the tubular basement membranes with most of them lacking LC restriction in proximal tubules and/or being associated with MIDD, amyloidosis, or cast-NP and, therefore, being excluded. One case meeting no other exclusion criteria (9) showed only very mild interstitial inflammation and no tubulitis, so that in this case, criteria were also not fulfilled (data not shown). Our constipative2 group was first recognized as a separate group on the basis of its morphologic features reminiscent of protein overload in high-proteinuric glomerular disease and then distinguished from the other groups with LC-R/C on the basis of its association with AL amyloidosis and nephrotic syndrome. Atypical lysosomes and crystal-like inclusions in an earlier study were restricted to LCPT (18). As in earlier reports, crystalline inclusions in LC-R/C showed a variety of shapes and substructures and could be missed by light microscopy (9, 10, 12). Toluidine blue-stained semithin sections were very helpful for detecting crystals (11, 19). One crystalline LC-R/C after antigen retrieval by heating showed an atypical staining pattern with negativity of constipated epithelia in both the LC stainings, which also occurs after antigen retrieval with pronase digestion (9, 12). Therefore, antigen retrieval of formalin-fixed paraffin-embedded (FFPE) material by heating appeared equivalent to previously described methods. Two cases of crystalline LC-R/C would have been missed without EM, as they showed no LC-R. The importance of ultrastructural analyses in detecting LCPT has been previously reported (35). Both cases showed no or only mild sign of ATI and one case was associated with normal renal function, so that one could question whether this finding is of “renal significance.” As we could not perform immunogold labeling, we could formally not proof that the crystals were derived from LC.

An ultrastructural finding exclusively found in the group of LC-R/C was the presence of “cloudy lysosomes,” which might, thus, be indicative of intracytoplasmic accumulation of LC. It has been reported that LC in LCPT can deposit as fibrils (9, 35, 36). Fibrils were not specifically associated with LC-R/C in our analyses, so that it appears that this finding can be associated with LC deposition, but it is not a proof. In an early report of changes of proximal tubules in LC-related disease, atypical lysosomes were described (18). Moreover, lysosomes with mottled appearance have been shown in the context of LCPT (11, 12, 35, 37). In our cohort, lysosomes with mottled appearance were by no means specific of LC-R/C and were even more prevalent in controls. Moreover, the shape and size of lysosomes or myelin bodies were not helpful in detecting LC-R/C.

In conclusion, LC-R/C is a prevalent finding in the context of MG- or MM-induced renal changes. However, it is not clear whether this finding is of “renal significance” *per se* in a proportion of cases. Some evidence indicates that it is often a sign of “trafficking” in the context of high, monoclonal LC loads rather than an independent disease process. However, LC-R/C was frequently associated with another LC-induced nephropathy warranting cytoreductive therapy. In the remainder of cases, decision regarding therapy indication probably should be made on a case-by-case basis, as suggested earlier (7). The frequent association with other diseases and a previous observation that LCPT can precede another LC-induced nephropathy advocate close follow-up of patients with LC-R/C, in order not to miss the development of a renal lesion of significance that requires therapy.

## Data Availability Statement

The original contributions presented in the study are included in the article/Supplementary Material, further inquiries can be directed to the corresponding author.

## Ethics Statement

The studies involving human participants were reviewed and approved by Ethics Committee of Friedrich-Alexander-University Erlangen-Nürnberg (Reference No. 4415). Written informed consent for participation was not required for this study in accordance with the national legislation and the institutional requirements.

## Author Contributions

MB-H designed the study, performed pathological evaluations and statistical analyses, and wrote the manuscript. NK performed *in vitro* experiments. TC collected the clinical data. KM participated in the establishment of the cohort and the collection of clinical data. FP participated in critical discussion of histological findings. CD participated in collection of data and statistical analyses. MK made micrographs of ultrathin EM sections. AB and FF participated in performing statistical analyses. KA participated in critical discussion of histological findings. All authors provided significant intellectual input and approved the final version of the manuscript.

## Conflict of Interest

The authors declare that the research was conducted in the absence of any commercial or financial relationships that could be construed as a potential conflict of interest.

## Publisher’s Note

All claims expressed in this article are solely those of the authors and do not necessarily represent those of their affiliated organizations, or those of the publisher, the editors and the reviewers. Any product that may be evaluated in this article, or claim that may be made by its manufacturer, is not guaranteed or endorsed by the publisher.

## References

[1] Dispenzieri A Katzmann JA Kyle RA Larson DR Melton LJ III Colby CL Prevalence and risk of progression of light-chain monoclonal gammopathy of undetermined significance: a retrospective population-based cohort study. *Lancet.* (2010) 375:1721–8. 10.1016/s0140-6736(10)60482-5 20472173 PMC2904571

[2] Stratta P Gravellone L Cena T Rossi D Gaidano G Fenoglio R Renal outcome and monoclonal immunoglobulin deposition disease in 289 old patients with blood cell dyscrasias: a single center experience. *Crit Rev Oncol Hematol.* (2011) 79:31–42. 10.1016/j.critrevonc.2010.05.001 20570173

[3] Sayed RH Wechalekar AD Gilbertson JA Bass P Mahmood S Sachchithanantham S Natural history and outcome of light chain deposition disease. *Blood.* (2015) 126:2805–10. 10.1182/blood-2015-07-658872 26392598 PMC4732758

[4] Hutchison CA Cockwell P Stringer S Bradwell A Cook M Gertz MA Early reduction of serum-free light chains associates with renal recovery in myeloma kidney. *J Am Soc Nephrol.* (2011) 22:1129–36. 10.1681/asn.2010080857 21511832 PMC3103732

[5] Hutchison CA Heyne N Airia P Schindler R Zickler D Cook M Immunoglobulin free light chain levels and recovery from myeloma kidney on treatment with chemotherapy and high cut-off haemodialysis. *Nephrol Dial Transplant.* (2012) 27:3823–8. 10.1093/ndt/gfr773 22273664

[6] Doshi M Lahoti A Danesh FR Batuman V Sanders PW. Paraprotein-related kidney disease: kidney injury from paraproteins-what determines the site of injury? *Clin J Am Soc Nephrol.* (2016) 11:2288–94. 10.2215/cjn.02560316 27526707 PMC5142058

[7] Sethi S Fervenza FC Rajkumar SV. Spectrum of manifestations of monoclonal gammopathy-associated renal lesions. *Curr Opin Nephrol Hypertens.* (2016) 25:127–37. 10.1097/MNH.0000000000000201 26735145

[8] Herrera GA Joseph L Gu X Hough A Barlogie B. Renal pathologic spectrum in an autopsy series of patients with plasma cell dyscrasia. *Arch Pathol Lab Med.* (2004) 128:875–9. 10.1043/1543-216520041282.0.CO;2 15270616

[9] Herrera GA. Proximal tubulopathies associated with monoclonal light chains: the spectrum of clinicopathologic manifestations and molecular pathogenesis. *Arch Pathol Lab Med.* (2014) 138:1365–80. 10.5858/arpa.2013-0493-OA 25268200

[10] Kapur U Barton K Fresco R Leehey DJ Picken MM. Expanding the pathologic spectrum of immunoglobulin light chain proximal tubulopathy. *Arch Pathol Lab Med.* (2007) 131:1368–72. 10.5858/2007-131-1368-ETPSOI 17824791

[11] Larsen CP Bell JM Harris AA Messias NC Wang YH Walker PD. The morphologic spectrum and clinical significance of light chain proximal tubulopathy with and without crystal formation. *Mod Pathol.* (2011) 24:1462–9. 10.1038/modpathol.2011.104 21701535

[12] Stokes MB Valeri AM Herlitz L Khan AM Siegel DS Markowitz GS Light chain proximal tubulopathy: clinical and pathologic characteristics in the modern treatment era. *J Am Soc Nephrol.* (2016) 27:1555–65. 10.1681/ASN.2015020185 26374607 PMC4849818

[13] Bridoux F Leung N Hutchison CA Touchard G Sethi S Fermand JP Diagnosis of monoclonal gammopathy of renal significance. *Kidney Int.* (2015) 87:698–711. 10.1038/ki.2014.408 25607108

[14] Beasley TM Schumacker RE. Multiple regression approach to analyzing contingency tables: post hoc and planned comparison procedures. *J Exp Educ.* (1995) 64:79–93. 10.1080/00220973.1995.9943797

[15] Sanders PW. Mechanisms of light chain injury along the tubular nephron. *J Am Soc Nephrol.* (2012) 23:1777–81. 10.1681/ASN.2012040388 22997259

[16] Batuman V. Proximal tubular injury in myeloma. *Contrib Nephrol.* (2007) 153:87–104. 10.1159/000096762 17075225

[17] Leboulleux M Lelongt B Mougenot B Touchard G Makdassi R Rocca A Protease resistance and binding of Ig light chains in myeloma-associated tubulopathies. *Kidney Int.* (1995) 48:72–9. 10.1038/ki.1995.269 7564094

[18] Sanders PW Herrera GA Lott RL Galla JH. Morphologic alterations of the proximal tubules in light chain-related renal disease. *Kidney Int.* (1988) 33:881–9. 10.1038/ki.1988.80 3133519

[19] Messiaen T Deret S Mougenot B Bridoux F Dequiedt P Dion JJ Adult Fanconi syndrome secondary to light chain gammopathy. Clinicopathologic heterogeneity and unusual features in 11 patients. *Medicine (Baltimore).* (2000) 79:135–54. 10.1097/00005792-200005000-00002 10844934

[20] Sharma SG. Light chain proximal tubulopathy: expanding the pathologic spectrum with and without deposition of crystalline inclusions. *ISRN Pathol.* (2012) 2012:1–6. 10.5402/2012/541075

[21] Lerner G Moradi S Cohen-Bucay A Chen H Sanchorawala V Gordon CE Coincidental crystalline light chain cast nephropathy, light chain proximal tubulopathy, and urine crystallopathy: a case report and review of the literature. *Clin Nephrol.* (2020) 93:203–8. 10.5414/cn109770 31907143

[22] Maldonado JE Velosa JA Kyle RA Wagoner RD Holley KE Salassa RM. Fanconi syndrome in adults. A manifestation of a latent form of myeloma. *Am J Med.* (1975) 58:354–64. 10.1016/0002-9343(75)90601-4 163583

[23] Leung N Bridoux F Batuman V Chaidos A Cockwell P D’Agati VD The evaluation of monoclonal gammopathy of renal significance: a consensus report of the international kidney and monoclonal gammopathy research group. *Nat Rev Nephrol.* (2019) 15:45–59. 10.1038/s41581-018-0077-4 30510265 PMC7136169

[24] Leung N Gertz M Kyle RA Fervenza FC Irazabal MV Eirin A Urinary albumin excretion patterns of patients with cast nephropathy and other monoclonal gammopathy-related kidney diseases. *Clin J Am Soc Nephrol.* (2012) 7:1964–8. 10.2215/CJN.11161111 23024162 PMC3513751

[25] Finkel KW Cohen EP Shirali A Abudayyeh A American Society of Nephrology Onco-Nephrology Forum. Paraprotein-related kidney disease: evaluation and treatment of myeloma cast nephropathy. *Clin J Am Soc Nephrol.* (2016) 11:2273–9. 10.2215/CJN.01640216 27526708 PMC5142056

[26] Nasr SH Valeri AM Sethi S Fidler ME Cornell LD Gertz MA Clinicopathologic correlations in multiple myeloma: a case series of 190 patients with kidney biopsies. *Am J Kidney Dis.* (2012) 59:786–94. 10.1053/j.ajkd.2011.12.028 22417785

[27] Sethi S Rajkumar SV D’Agati VD. The complexity and heterogeneity of monoclonal immunoglobulin-associated renal diseases. *J Am Soc Nephrol.* (2018) 29:1810–23. 10.1681/ASN.2017121319 29703839 PMC6050917

[28] van de Donk NW Palumbo A Johnsen HE Engelhardt M Gay F Gregersen H The clinical relevance and management of monoclonal gammopathy of undetermined significance and related disorders: recommendations from the European myeloma network. *Haematologica.* (2014) 99:984–96. 10.3324/haematol.2013.100552 24658815 PMC4040895

[29] Herrera GA. Renal manifestations of plasma cell dyscrasias: an appraisal from the patients’ bedside to the research laboratory. *Ann Diagn Pathol.* (2000) 4:174–200. 10.1016/s1092-9134(00)90042-x 10919389

[30] Batuman V Guan S O’Donovan R Puschett JB. Effect of myeloma light chains on phosphate and glucose transport in renal proximal tubule cells. *Ren Physiol Biochem.* (1994) 17:294–300. 10.1159/000173861 7533308

[31] Hutchison CA Batuman V Behrens J Bridoux F Sirac C Dispenzieri A The pathogenesis and diagnosis of acute kidney injury in multiple myeloma. *Nat Rev Nephrol.* (2011) 8:43–51. 10.1038/nrneph.2011.168 22045243 PMC3375610

[32] Solomon A Weiss DT Kattine AA. Nephrotoxic potential of Bence Jones proteins. *N Engl J Med.* (1991) 324:1845–51. 10.1056/NEJM199106273242603 1904132

[33] Fermand JP Bridoux F Kyle RA Kastritis E Weiss BM Cook MA How I treat monoclonal gammopathy of renal significance (MGRS). *Blood.* (2013) 122:3583–90. 10.1182/blood-2013-05-495929 24108460

[34] Cheng M Gu X Turbat-Herrera EA Herrera GA. Tubular injury and dendritic cell activation are integral components of light chain-associated acute tubulointerstitial nephritis. *Arch Pathol Lab Med.* (2019) 143:1212–24. 10.5858/arpa.2018-0032-OA 31063013

[35] Brealey JK Tran Y Ninnes R Abeyaratne A. Ultrastructural identification of a proximal tubulopathy without crystals in a relapsed multiple myeloma patient. *Ultrastruct Pathol.* (2018) 42:458–63. 10.1080/01913123.2018.1526243 30252563

[36] Yao Y Wang SX Zhang YK Wang Y Liu L Liu G. Acquired Fanconi syndrome with proximal tubular cytoplasmic fibrillary inclusions of lambda light chain restriction. *Intern Med.* (2014) 53:121–4. 10.2169/internalmedicine.53.0836 24429451

[37] Jung M Lee Y Lee H Moon KC. Clinicopathological characteristics of light chain proximal tubulopathy in Korean patients and the diagnostic usefulness of immunohistochemical staining for immunoglobulin light chain. *BMC Nephrol.* (2020) 21:146. 10.1186/s12882-020-01813-w 32326898 PMC7178968

