# Supplementary material for: Light Chain Restriction in Proximal Tubules—Implications for Light Chain Proximal Tubulopathy
Source: Front Med (Lausanne). 2022 Mar 28;9:723758. doi: 10.3389/fmed.2022.723758 (PMC8995435; doi:10.3389/fmed.2022.723758)
Supplement: Supplementary file 1 [file Table_1.DOCX]

Supplementary Table 1:

Inclusion criteria, clinical categories, proteinuria and hematuria scores

| Inclusion criteria | Definition |
| --- | --- |
| Clinical |  |
| Monoclonal gammopathy (MG) | - known MG or MGUS - increased free LC kappa or lambda in serum or urine - pathological LC quotient in serum (<0.26, >1.65) or urine (<0.461, >4.00) - suspicion of MM - M/extra-spike - Bence-Jones proteinuria |
| Multiple myeloma (MM) | - Known MM - Known smoldering MM |
| Mature B-cell Non-Hodgkin lymphoma (BNHL) | - Known mature BNHL |
| Pathological |  |
| LC-induced nephropathy | - Cast-NP - MIDD - AL-amyloidosis - infiltration by MM or mature BNHL - immunotactoid glomerulonephritis - light microscopic diagnosis of LCPT |
| Umbrella term |  |
| B-cell dyscrasia | - including all of the above-mentioned findings |

Proteinuria score

| Score | 0 | 1 | 2 | 3 | 4 |
| --- | --- | --- | --- | --- | --- |
| [semiquantitative] | 0 | / | + | ++ | +++ |
| [g/l] | < 0.1 | 0.1 – 0.25 | 0.25 – 1.0 | 1.0 – 3.0 | > 3.0 |
| [g/d] | < 0.15 | 0.15 – 0.3 | 0.3 – 1.5 | 1.5 – 3.5 | > 3.5 |
| [g/g creatinine] | < 0.15 | 0.15 – 0.3 | 0.3 – 1.5 | 1.5 – 3.5 | > 3.5 |

Hematuria score

| Score | 0 | 1 | 2 | 3 |
| --- | --- | --- | --- | --- |
| [semiquantitative] | 0 | + | ++ | +++ |
| [erythrocytes/ high power field] | < 3 | 3 - < 20 | 20 - 50 | > 50 |
| [erythrocytes/µl] | < 5 | 5 - < 80 | 80 - 200 | > 200 |
